# Pandemics, public health emergencies and antimicrobial resistance - putting the threat in an epidemiologic and risk analysis context

**DOI:** 10.1186/s13690-017-0223-7

**Published:** 2017-09-14

**Authors:** C. Raina MacIntyre, Chau Minh Bui

**Affiliations:** 1 University of New South Wales, Sydney, NSW Australia; 2 Arizona State University, Phoenix, AZ USA

**Keywords:** Antimicrobial resistance, AMR, Superbug, Risk, Pandemic, Influenza, Bacterial infections, Disease burden, Genetic engineered pathogens, Public health

## Abstract

Public health messaging about antimicrobial resistance (AMR) sometimes conveys the problem as an epidemic. We outline why AMR is a serious endemic problem manifested in hospital and community-acquired infections.

AMR is not an epidemic condition, but may complicate epidemics, which are characterised by sudden societal impact due to rapid rise in cases over a short timescale. Influenza, which causes direct viral effects, or secondary bacterial complications is the most likely cause of an epidemic or pandemic where AMR may be a problem. We discuss other possible causes of a pandemic with AMR, and present a risk assessment formula to estimate the impact of AMR during a pandemic. Finally, we flag the potential impact of genetic engineering of pathogens on global risk and how this could radically change the epidemiology of AMR as we know it.

Understanding the epidemiology of AMR is key to successfully addressing the problem. AMR is an endemic condition but can play a role in epidemics or pandemics, and we present a risk analysis method for assessing the impact of AMR in a pandemic.

## Background

Antimicrobial resistance (AMR) is the resistance of bacteria, viruses or other micro-organisms to antimicrobial drugs. The most common and concerning form of AMR is antibiotic resistance against important bacteria. Many multi-resistant bacteria have emerged over the past century. AMR is driven by selective evolutionary pressure, and relates to the volume of antibiotic use through over-use or misuse in humans, animals and food production. AMR can also occur through random genetic mutations. Efforts to control antibiotic use have focused on human and animal health. AMR is an increasing concern globally, and can increase the overall impact of any infection by reducing or removing the ability to treat infections successfully. In this scenario the rate of illness and death would be the same as that seen in untreated infection.

## Main text

### Is AMR an epidemic?

**Fig. 1:**
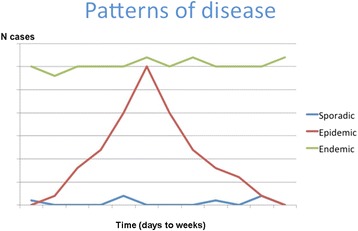
Patterns of disease

Epidemic and pandemic diseases must be differentiated from endemic diseases, such as malaria and tuberculosis (TB), which can exist in high numbers in a population and cause high burden of disease, but with a constant rate or slow change in rates. A true epidemic has a rise in case rates over days or weeks, whereas an endemic disease may rise or fall over years or decades. The words epidemic and pandemic are often misused. A pandemic is simply a global epidemic, and an epidemic is an outbreak of disease that attacks many people at about the same time and spreads through a defined population. It is defined by rate of growth of the epidemic curve. Epidemics have a sudden and immediate impact on the health system and require surge capacity. For transmissible diseases, epidemics have a mathematical definition related to the reproductive number, R_0_. A R_0_ of 1 is the epidemic threshold, and any R value greater than 1 presents necessary conditions for an epidemic. The rapid increase in cases observed during an epidemic or pandemic is what causes immediate impacts on health systems, stressing these systems beyond normal operating capacity. A pandemic of a highly infectious microbe will evolve rapidly and affect many people within a short period of time. For example, an influenza pandemic once it begins, would be expected to reach its peak within 2 months. A working group commissioned by the World Health Organization (WHO) has listed diseases with pandemic potential, but some of these, such as malaria, TB and AMR organisms, are usually endemic diseases [1]. Endemic diseases may occur in very high numbers, but their rate is constant or changes slowly, over months or years, sometimes decades. Sporadic diseases occur at a rate too low to be endemic or epidemic. They are typically zoonotic infections that occasionally infect humans. Figure 1 shows the difference in the three main patterns of disease – epidemic, endemic and sporadic.

AMR has been a chronic, slowly increasing phenomenon worldwide, and causes long term impact on the health system, as well as severe, immediate impact on affected individuals. Hospital infections such as Methicillin-resistant *Staphylococcus aureus* (MRSA) and multidrug resistant TB are well known examples, but increasingly common causes of urine infection and pneumonia are also becoming resistant. AMR is not an epidemic, but an endemic condition.

### AMR complicating an epidemic or pandemic

**Fig. 2:**
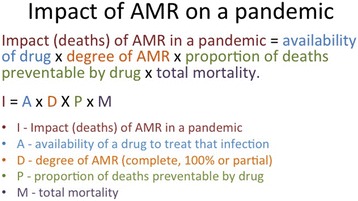
Impact of AMR on a pandemic

However, AMR can complicate an epidemic or pandemic. In the case of a pandemic, where large scale infections would affect the population in a short space of time, AMR could contribute adversely to serious illness and death on a mass scale. The mortality impact of AMR on a pandemic of any infection depends on a series of factors. Whilst there are other contributors, the main determinants are whether or not there is a drug available to treat the infection. If this is zero, then the impact of AMR is zero. It also depends on: the degree of AMR – whether it is partial, or complete; the total mortality caused by the infection; and the proportion of deaths preventable by that drug. These factors together, determine the impact of AMR on a pandemic. The equation can also be modified for morbidity. Many emerging infections (such as Ebola and MERS) have no available drug for treatment, so the impact of AMR is zero. The overall mortality impact of AMR can be summarised by Fig. 2. This equation is adapted from the “risk triangle” concept [2] which is widely used in disaster management, whereby ‘risk’ refers to potential lives lost and is proportionally dependent on three components: hazard, exposure and vulnerability.

Influenza is the most likely cause of a pandemic where AMR would be a factor. Influenza morbidity and mortality can be due to the virus itself or to secondary bacterial infections, therefore AMR can doubly impact an influenza pandemic through both antiviral and antibiotic resistance. Based on data from the 2009 influenza A(H1N1) pandemic, the case fatality rate could be anything from 1 to 20% in the absence of substantial AMR [3]. Bacterial infections can contribute to up to 50% of the total deaths [4], so if bacterial AMR is a factor, then in the worst case scenario up to 50% of deaths could not be prevented.

### The burden of disease of AMR

AMR may be primary or secondary. Primary AMR occurs when the infecting microbe is resistant at the outset and secondary AMR is when resistance develops during treatment, which may occur when people do not finish a full course of antibiotics or when antibiotics are used inappropriately. AMR infections may arise in a surgical wound, at the site of an intravenous cannula, or other skin surfaces which have been breached due to a medical procedure. Resistant infections can also arise in the bowel, urine or the lungs (for example, ventilator associated pneumonia) and are a risk in severely ill patients in intensive care. The impact of hospital acquired AMR is serious for the individual patient and can result in severe medical complications, extended hospital length of stay and death. Hospital acquired infections contribute significantly to avoidable illness and death. In the United States (US) over 2 million people acquire an infection from a hospital each year, with around 70% of these hospital-acquired infections caused by AMR strains [5]. Those who are at highest risk for hospital-acquired AMR infections are those in intensive care units (ICUs), chronic care areas, or nursing homes and those who are immunocompromised. Community-acquired resistance is an increasing problem with examples being urinary tract infections and pneumonia. In its endemic state, these are the manifestations of AMR, which contributes to chronic burden of disease.

A 2014 review paper on AMR [6] estimated up to 50,000 deaths in Europe and the US alone are attributed to AMR, and globally, AMR causes around 700,000 deaths annually – these figures consider AMR bacterial infections, malaria, human immunodeficiency virus/acquired immunodeficiency syndrome (HIV/AIDS) and TB. The review also predicts that by the year 2050, AMR will cause around 10 million deaths annually. The reliability of these estimates (i.e. of 10 million deaths by 2050) have been questioned as an imprecise over-estimate which draws on biased sources of data from hospital-based surveillance systems, which do not reflect community burden of disease [7]. Further, the scenarios from which these estimates were generated are unrealistic as they assume a rise in resistance levels for all bacteria, and that the rate of people becoming infected will increase while the mortality risk per infection remains the same [7]. Current evidence suggests these scenarios are unlikely because mortality rates associated with bacterial infections are actually decreasing because of increasing capability to provide appropriate hospital care. Furthermore, the methods used in the 2014 review were not been subject to peer review or scientific scrutiny and the report does not acknowledge levels of uncertainties associated with the estimates [7]. Finally, the global burden of disease study does not feature AMR among its leading causes of death and disability, and shows that since 2008 infections have been overtaken by non-communicable diseases globally as the leading cause of disease burden [8]. Respiratory infections, TB, malaria and HIV are the main infectious diseases in the top 10, and there has been a reduction globally in communicable diseases. Whilst multi-drug resistant TB (MDRTB) is increasing, less than 4% of TB is MDRTB globally, and HIV drug resistance is falling in many countries. Many of the respiratory infections which contribute to global burden of disease occur in countries where access to antimicrobials is uncommon.

AMR may cause acute burden of disease in a population during a pandemic, depending on various factors. These include the ability of the microbe to spread rapidly and cause a pandemic or large epidemic (for example, some common types of pneumonia which may complicate influenza are not transmissible between people); the severity of the disease caused by the microbe; the degree of AMR (partial or complete); and the number of drugs to which resistance is present – multidrug resistant strains have a greater impact. In addition, whether there are alternative drugs to use in place of the ineffective drug and the extent which disease control efforts rely on the use of antimicrobials to mitigate a pandemic will also have an impact.

These factors are influenced by inherent characteristics of the pathogens themselves in addition to external contextual factors. For example, the ability of the microbe to spread is determined by epidemiological parameters including R_0_, incubation period and transmission rates. The burden of AMR during a pandemic will also vary for different regions – for example, low income regions will likely suffer a higher burden due to poorer health care standards in hospitals, greater crowding, limited access to laboratories, widespread purchasing of antimicrobials without prescription and poor regulatory frameworks for antibiotic use.

### Specific diseases with pandemic and AMR potential

#### Influenza

Of the diseases that have potential to cause a pandemic where AMR may be a contributing factor to health impact, influenza is the most likely. Influenza has potential to cause large scale morbidity and mortality over a relatively short period of time due to rapid spread between people, and there are commercially available antiviral drugs currently available for use in the event of an influenza pandemic. Antivirals will be heavily relied upon for control of a pandemic in the early phase, as the development of a vaccine matched to the pandemic influenza strain will take up to 6 months. In 2003, adamantane-resistant H3N2 influenza strains emerged, rendering adamantanes ineffective for treatment. The main class of antivirals available presently are the neuraminidase inhibitors, including oseltamivir and zanamivir. Dual adamantane-oseltamivir resistant strains have been reported sporadically in seasonal H1N1 in Cambodia (2007) and Hong Kong (2008) but are not considered to be widespread.

In 2007 oseltamivir-resistant seasonal influenza H1N1 (A/Brisbane/59/07) strains emerged as primary resistance in Europe. Unlike other anti-viral resistant strains of influenza, this particular virus strain was able to transmit efficiently from person to person. Within a year, the strain had spread globally, including in regions where oseltamivir was not used. Following the emergence of the H1N1pdm09 virus in 2009, the oseltamivir-resistant seasonal influenza H1N1 (A/Brisbane/59/07) strains died out, and strains circulating across the globe since then have been largely sensitive to NAIs. The fact that only a small number of oseltamivir resistance H1N1pdm09 viruses have been found so far, suggests that resistant strains may have reduced ability to transmit from person to person, and hence may not interfere with the efficacy of high levels of antiviral usage. The largest documented community outbreak of oseltamivir-resistant H1N1pdm09 strains was identified in Australia in 2011, and involved only 29 people [9]. Clinical outcomes for those in this cluster typically resulted in influenza-like-illnesses, but severe disease and fatalities were not reported [9].To date, influenza strains resistant to oseltamivir and zanamivir have been rare, with most reports occurring in individuals with immunodeficiency.

Death and disease from pandemic influenza may be due to the virus itself, or from secondary bacterial infections which cause pneumonia and sepsis. Therefore bacterial antibiotic resistance may also contribute to overall impact. The most common bacterial infection in a pandemic is *Streptococcus pneumoniae* – the percentage of bacteria resistant to penicillin varies widely according to resistance definitions (from <1% to up to 9.4% in one study [10]) and region [11], and up to 25% in other studies [12]. There are effective vaccines against *Streptococcus pneumoniae*, funded for people >65 years in many countries.

Bacterial coinfections have been reported to play a role in around a third of influenza cases managed in ICUs and up to 30–50% of fatalities are attributed to bacterial infections [4]. Other bacteria commonly causing in secondary infections include *Staphylococcus aureus* and *Haemophilus influenzae*. AMR is a significant concern as community-acquired MRSA have been reported to cause complications in seasonal influenza seasons and during the 2009 pandemic. In a sample of 838 critically ill minors during the 2009 influenza pandemic in the US, 33% had evidence of bacterial confection, of these, 39% were attributed to *Staphylococcus aureus,* and among these 58% were methicillin-resistant [13]. During the 2003–2004 influenza season in the US, there were 18 people reported to have severe secondary bacterial infections associated with MSRA. Of these 18 people, the median age was 21 years, five had an underlying illness, 81% required intensive care, 62% required positive pressure ventilation and 29% died [5, 14].

Other multi-drug resistant bacteria with the potential to impact morbidity and mortality during an influenza pandemic especially in ventilated ICU patients include (i) those which cause acquired AMR such as vancomycin resistant *Enterococcus* (VRE), *Pseudomonas*, *Acinetobacter* and drug resistant pneumococci, and (ii) those which cause intrinsic AMR such as *Clostridium difficile*.

#### Plague

Past pandemics have been caused by the bubonic plague, caused by the bacterium *Yersinia pestis* which often infects small rodents and is transmitted to humans through the bite of an infected flea. However this disease is unlikely to occur in high income settings which have good sanitation. Historical pandemics caused by the plague include the ‘the Justinian Plague’ of 541 AD, the ‘Black Death’ which emerged in China in 1334, and the ‘Modern Plague’ which began in China in the 1860s. Large scale epidemics of plague are unlikely to occur in countries where there are no animal reservoirs for *Y. pestis*. Whilst plague is an unlikely cause of a pandemic, it can be treated with antibiotics, therefore AMR would substantially increase the fatality rate of the disease.

#### Smallpox

Smallpox is caused by the variola virus and was globally eradicated in 1980 following a vaccination campaign led by the WHO. Small pox is contagious and spreads through respiratory droplets from infected cases as well as contact. Smallpox is only transmissible while patients are symptomatic. It does not usually cause very large outbreaks and spread usually occurs to members living in the same household. Large outbreaks in schools were not commonly reported. Fatalities occur in up to 30% of cases (with higher case fatality rates in high risk groups such as children less than a year and in immunosuppressed individuals). The smallpox virus officially exists in just two high-security laboratories in Russia and the US for scientific research, hence there is still a risk of the smallpox virus escaping the laboratory and causing a pandemic. The genome of smallpox is also fully sequenced and known, so it is technically possible for smallpox to be synthetically engineered in a laboratory. Many governments procure and stockpile antivirals for use in the event of a smallpox outbreak – hence the development of AMR may be relevant in the event of a smallpox pandemic. However, little is known about the effectiveness of currently available antivirals such as cidofivir and tecovirimat. It is assumed they may be effective based on lab experiments, however, because these drugs were not tested in people with smallpox, it is not known how effective they will be. No antiviral resistant smallpox strains have been reported so far. Secondary bacterial infections of the skin can also occur as a complication of smallpox, and would be treated by penicillin and cephalosporin antibiotics. These would be more likely to be affected by AMR, but the net impact on morbidity and mortality is unknown. The equation provided above could be applied to a smallpox outbreak at the time, as real data becomes available, to estimate impact.

#### Other infections

In their natural state, diseases such as TB, malaria and HIV, whilst on the WHO list, are largely endemic diseases which occur in large numbers at a constant or slowly evolving rate in many populations. TB and HIV also have a long asymptomatic phase and in most cases onset of clinical disease years after initial infection. They do not, therefore, have a large, sudden population impact with clinical cases increasing rapidly over a short period of time. This could change if these microbes are artificially engineered for rapid spread or altered latent periods.

Severe acute respiratory syndrome (SARS) was an epidemic infection, in contrast to Middle East Respiratory Syndrome (MERS), which has displayed a more sporadic pattern with occasional outbreaks. For most emerging viruses which have spread globally (such as SARS, MERS, Ebola, dengue, encephalitis), there are no commercially available anti-virals, and thus AMR is not relevant. However, drugs are in development for some of these, such as Ebola, which has epidemic potential.

#### Genetic engineered pathogens

The discussion above focuses entirely on natural events. However, there is an increasing risk of unnatural pandemics due to quantum advances in science, availability of methods for engineering pathogens, open access science and global geopolitical instability. Unnatural pandemics may occur as a result of deliberate release or a laboratory accident. Our systems, governance and legislation have not kept pace with changes in science, leaving the world vulnerable. Developments in gene editing, particularly Clustered regularly interspaced short palindromic repeats (CRISPR) associated protein-9 nuclease (Cas9), makes it possible today to engineer plants, animals, human beings and microbes. Editing of healthy human embryos, for example, is underway in Sweden, and modification of avian influenza viruses, SARS and other pathogens has already occurred. Synthetic viruses and genetic engineering of pathogens is an example of dual-use research of concern (DURC) - research intended for benefit but which may also harm humans. The capacity for biological weapons of mass destruction is vastly greater today than even a decade ago, and an unnatural pandemic may not be correctly detected as unnatural because of our inability to differentiate unnatural from natural epidemics [15]. This caveat is provided to make the point that with new gene editing technology, highly lethal or resistant microbes can be created in a laboratory and released in populations. This may override our current knowledge of how microorganisms behave in nature, as their natural characteristics such as resistance profile, latent period, incubation period and transmissibility can be altered significantly.

## Conclusions

In summary, in its present form, AMR is a gradually increasing problem with a chronic and endemic pattern. The global concern about AMR can be broken down into two separate concerns. The first is the gradually rising incidence of AMR in many bacteria including TB and many hospital or community acquired infections. This is an endemic problem, driven by patterns of antibiotic use in animals and humans, with a gradual rise in cases over time. The impact on a population level can be compared to other endemic diseases such as diabetes or heart disease. The second concern is AMR as a compounding factor in a pandemic infection. Whilst the term pandemic is widely misused, technically it refers to a global epidemic in which there is a rapid rise in cases over time, and therefore an immediate, sharp impact on society and its systems. If AMR affected a pandemic, it would contribute to overall death and disability on a sudden and large scale. Of all currently known infections with pandemic potential, influenza is the most probable cause of a major human pandemic. There are other potential causes, but many of these have no available drugs for treatment. In addition, the technology exists today to change living organisms from their natural state into an unnatural state, which may result in previously unknown types of pandemics. The exact impact of AMR in a pandemic needs to be quantified for specific diseases, and we have provided a general formula which can be used to do so. This formula can be expanded with many more factors, but the most important determinants of impact are included.

## Abbreviations

AIDS: Acquired immunodeficiency syndrome
AMR: Antimicrobial resistance
CRISPR: Clustered regularly interspaced short palindromic repeats
CRISPR-cas9: CRISPR-associated protein-9 nuclease
DURC: Dual-use research of concern
HIV: Human immunodeficiency virus
ICU: Intensive Care Unit
MDRTB: Multi-drug resistant tuberculosis
MERS: Middle East Respiratory Syndrome
MRSA: Methicillin-resistant *Staphylococcus aureus*
SARS: Severe acute respiratory syndrome
TB: Tuberculosis
US: United States
WHO: World Health Organization

## References

[1] World Health Organization. Blueprint for R&D preparedness and response to public health emergencies due to highly infectious pathogens - Workshop on prioritization of pathogens (8–9 December 2015). http://www.who.int/medicines/ebola-treatment/WHO-list-of-top-emerging-diseases/en/. Accessed 29 Nov 2016.

[2] Chrichton D. The Risk Triangle. In: Natural Disaster Management. edn. Edited by Ingleton J. Tudor Rose, London; 1999.

[3] Joseph C, Togawa Y, Shindo N (2013). Bacterial and viral infections associated with influenza. Influenza Other Respir Viruses.

[4] Papanicolaou GA (2013). Severe influenza and S. Aureus pneumonia: for whom the bell tolls?. Virulence.

[5] Memoli MJ, Morens DM, Taubenberger JK (2008). Pandemic and seasonal influenza: therapeutic challenges. Drug Discov Today.

[6] The Review on Antimicrobial Resistance. Antimicrobial resistace: Tackling a crisis for the health and wealth of nations (December 2014). https://tinyurl.com/zmylsav. Accessed 3 Mar 2017.

[7] de Kraker ME, Stewardson AJ, Harbarth S (2016). Will 10 million people die a year due to antimicrobial resistance by 2050?. PLoS Med.

[8] World Health Organisation (WHO). Health statistics and information systems, Estimates for 2000–2015: Cause-specific mortality. http://www.who.int/healthinfo/global_burden_disease/estimates/en/index1.html. Accessed 20 Mar 2017.

[9] Hurt AC, Hardie K, Wilson NJ, Deng YM, Osbourn M, Leang SK, Lee RT, Iannello P, Gehrig N, Shaw R (2012). Characteristics of a widespread community cluster of H275Y oseltamivir-resistant a(H1N1)pdm09 influenza in Australia. J Infect Dis.

[10] Goossens MC, Catry B, Verhaegen J (2013). Antimicrobial resistance to benzylpenicillin in invasive pneumococcal disease in Belgium, 2003–2010: the effect of altering clinical breakpoints. Epidemiol Infect.

[11] Linares J, Ardanuy C, Pallares R, Fenoll A (2010). Changes in antimicrobial resistance, serotypes and genotypes in Streptococcus Pneumoniae over a 30-year period. Clin Microbiol Infect.

[12] Appelbaum PC (2002). Resistance among Streptococcus Pneumoniae: implications for drug selection. Clin Infect Dis.

[13] Randolph AG, Vaughn F, Sullivan R, Rubinson L, Thompson BT, Yoon G, Smoot E, Rice TW, Loftis LL, Helfaer M (2011). Critically ill children during the 2009–2010 influenza pandemic in the United States. Pediatrics.

[14] Hageman JC, Uyeki TM, Francis JS, Jernigan DB, Wheeler JG, Bridges CB, Barenkamp SJ, Sievert DM, Srinivasan A, Doherty MC (2006). Severe community-acquired pneumonia due to Staphylococcus Aureus, 2003–04 influenza season. Emerg Infect Dis.

[15] MacIntyre, CR. Biopreparedness in the age of genetically engineered pathogens and open access science - an urgent need for a paradigm shift. Military Medicine. 2015;180:943–9. 10.7205/MILMED-D-14-00482 PMC7107569 26327545

